# Atypical presentation of Charcot-Marie-Tooth disease type 1C with a new mutation: a case report

**DOI:** 10.1186/s12883-021-02316-3

**Published:** 2021-07-27

**Authors:** Monika Turčanová Koprušáková, Milan Grofik, Ema Kantorová, Petra Jungová, Ján Chandoga, Martin Kolisek, Peter Valkovič, Matej Škorvánek, Rafal Ploski, Egon Kurča, Štefan Sivák

**Affiliations:** 1 Clinic of Neurology, Jessenius Faculty of Medicine in Martin, Comenius University in Bratislava, Kollárova 2, 036 01 Martin, Slovak Republic; 2 Institute of Medical Biology, Genetics and Clinical Genetics, Faculty of Medicine, Comenius University and University Hospital in Bratislava, Bratislava, Slovak Republic; 3 Biomedical Center Martin, Jessenius Faculty of Medicine in Martin, Comenius University in Bratislava, Mala Hora 4b, 036 01 Martin, Slovak Republic; 4 2nd Department of Neurology, Comenius University and University Hospital in Bratislava, Bratislava, Slovak Republic; 5 Centre of Experimental Medicine, Institute of Normal and Pathological Physiology, Slovak Academy of Sciences, Slovak, Slovak Republic; 6 Department of Neurology, P.J. Safarik University and Louis Pasteur University Hospital, Kosice, Slovak Republic; 7 Department of Medical Genetics Laboratory, Medical University of Warsaw, Warsaw, Poland

**Keywords:** Charcot-Marie-Tooth 1C, LITAF, Inflammatory neuropathy, Case report

## Abstract

**Background:**

Charcot-Marie-Tooth 1C (CMT1C) is a rare form of dominantly inherited CMT1 neuropathy caused by a mutated gene encoding lipopolysaccharide-induced tumour necrosis alpha factor (LITAF).

**Case presentation:**

We report a 56-year-old patient with an atypical clinical phenotype of CMT1C, which started as progressive weakness of a single upper limb resembling acquired inflammatory neuropathy. Nerve conduction studies (NCS) and temporarily limited and partial effects of immunotherapy supported the diagnosis of inflammatory neuropathy. Significant progression of polyneuropathy, despite intensive long-lasting immunotherapy, together with repeatedly negative auxiliary investigations (CSF, MRI and antibodies) and genetic testing results finally led to the diagnosis of CMT1C neuropathy.

**Conclusions:**

CMT1C should be added to the list of inherited neuropathies that need to be considered in suspected cases of inflammatory demyelinating neuropathy.

## Background

Charcot-Marie-Tooth 1C (CMT1C) is an autosomal dominant demyelinating peripheral neuropathy associated with mutations in the LITAF (lipopolysaccharide-induced tumour necrosis alpha factor) gene. Mutations in LITAF are very rare (0.5–0.9%) [1, 2], and only a few cases with detailed clinical and electrophysiological descriptions have been reported. Therefore, data on the clinical phenotypes of CMT1C are very limited. CMT1C was described mostly as a mild or moderate form of slowly progressive symmetric length-dependent sensory-motor demyelinating polyneuropathy often associated with pes cavus foot deformity and bilateral foot drop [3]. Patients carrying LITAF mutations may present with either a classical CMT1A phenotype or a sensory phenotype limited to isolated paresthesias [4]. Recently an atypical course of CMT1C was described as slowly progressive bilateral calf pain and cramps [5].

## Case presentation

A 56-year-old man developed isolated paresthesias, weakness and cramps of the right upper limb over a period of three months. Previously he had been healthy with no history of long-term medications, engaging in variety of sports (cycling, running, cross-country skiing, bow-shooting). Neurological examination revealed isolated distal muscle weakness of the right upper limb (MRC 3), normal muscle strength of the left upper limb (MRC 5), generalized hyporeflexia and the presence of pes cavus bilaterally with normal muscle strength (MRC 5) and reduced vibration sense (ankle 4/8, toe 2/8) in the lower limbs. Slightly reduced pin prick and thermic sensation was detected. The patient was able to walk on heels and tiptoes. Mild ataxia when standing with eyes closed (positive Romberg sign) was also present. Chronic progression of muscle weakness followed during the next six months. The right upper limb showed increased distal weakness (wrist extensors MRC 3, finger extensors MRC 1, wrist and finger flexors MRC 3, intrinsic hand muscles MRC 3). Afterwards, involvement of the left upper limb with mild weakness and paresthesia also appeared (wrist extensors MRC 5, finger extensors MRC 3, wrist and finger flexors MRC 4, intrinsic hand muscles MRC 4). As a result, the patient had difficulties with fine motor tasks, such as buttoning or unbuttoning of clothes, unscrewing bottle caps and turning door-knobs. No weakness of proximal upper limb muscles was registered (MRC 5). Muscle strength in lower limbs was normal (MRC 5).

Nerve conduction studies (NCS) were consistent with moderate primary demyelinating sensory-motor polyneuropathy of the upper limbs with partial conduction blocks in some nerves (median, ulnar) and severe sensory-motor polyneuropathy of the lower limbs. Compound muscle action potentials (CMAPs) showed prolonged distal motor latencies, reduced nerve conduction velocities and low amplitudes with prolonged minimal F wave latencies. Sensory nerve action potentials (SNAPs) in the upper limbs showed prolonged distal latencies, reduced velocities and low amplitudes. SNAPs in lower limbs were absent (Table 1). Needle electromyography revealed a severe chronic denervation syndrome with sporadic spontaneous activity in upper limb muscles (biceps brachii, deltoideus, extensor digitorum communis, interosseus dorsalis primus, and abductor pollicis).

**Table 1:** Nerve conduction studies in patient II/5

Nerve	Nerve segment	Index patient II/5 (60) 11/2020				Index patient II/5 (58) 9/2018				Index patient II/5 (56) 1/2017			
**MNCS**		Lat ms	Amp mV	MCV m/s	Dur ms	Lat ms	Amp mV	MCV m/s	Dur ms	Lat ms	Amp mV	MCV m/s	Dur ms
**Median**													
Wrist	Wr-APB	7.5	0.1		6.5	6.9	0.8		6.4	6.1	2.5		6.2
Elbow	El-Wr	19.3	0.06	24.5	7.3	19.6	0.5	30.6	7.4	13.6	1.8	33	7.1
Axilla	Ax-El	27.8	0.06	30.6	7.5	28.4	0.4	-	7.9				
**Ulnar**													
Wrist	Wr-ADM	4.8	9.1		6.9	4.5	11		7.6	4.4	7.8		7.8
Elbow	BE-Wr	11.7	6.4	33.4	7.7	12.4	10	32.7	8.6	11.7	6.4	34.6	8.2
Axilla	AE-BE	15.8	5.1	30.3	8.7	15.2	9.1	35.7					
	Ax-BE	21.4	3.4	35.7	8.7	23.1	5.5	46					
**Radial**													
Forearm	For-EIP	3.6	1.6		7.3	NA				NA			
Elbow	CF-For	5.4	0.8	37.9	5.7								
Arm	Spir.g – CF	7.4	0.6	34.8	6.1								
**Peroneal**													
Ankle	An-EDB	7.9	0.15		4.5	NA				NA			
Fibula	FH-An	22.7	0.14	20.8	4.8								
FP	FP-FH	27.3	0.14	22.2	4.6								
**Tibial**													
Ankle	AH-An	5.9	0.18		3.8	NA				5.7	0.06	23.5	3.8
FP	An-FP	28.9	0.04	18.2	6.3					24.5	0.02		6.5
**SNCS**		Lat ms	Amp uV	SCV m/s		Lat ms	Amp uV	SCV m/s		Lat ms	Amp uV	SCV m/s	
**Median**	Wr-II	6.5	5.8	39.5		5.9	6.1	35.4		5.6	3.2	37.2	
**Ulnar**	Wr-V	5.6	4.9	30.8		5.0	6.3	32.3		4.9	5.7	34.4	
**Radial**	Wr-I	3.9	4.3	42.0		3.8	4.5	42.3		NA			
**Sural**	Calf-LM	NE				NE				NE			

Legend: *MNCS* Motor nerve conduction studies, *SNCS* Sensory nerve conduction studies, *Lat* Latency, *Amp* Amplitude, *Dur* Duration, *MCV* Motor conduction velocity, *SCV* Sensory conduction velocity, *AH* Abductor hallucis, *APB* Abductor pollicis brevis, *ADM* Abductor digiti minimi, *EDB* Extensor digitorum brevis, *EIP* Extensor indicis proprius, *Wr* Wrist, *AE* Above elbow, *BE* Below elbow, *For* Forearm, *Ax* Axilla, *CF* Cubital fossa, *Spir.g* Spiral groove, *An* Ankle, *FH* Fibular head, *FP* Fossa poplitea, *LM* Lateral malleolus, *NA* Not available, *NE* Not elicited, *ms* Millisecond, *uV* Microvolt, *m/s* Meter per second

Laboratory tests revealed normal values, including the absence of paraprotein. CK (creatine kinase) levels were two-fold greater than normal. Antineuronal, antiganglioside and anti-MAG (myelin-associated glycoprotein precursor) antibodies were negative. Nodal/paranodal antibodies (NF 155, 140/186, contactin 1 and Caspr) were also negative. Genetic testing for CMT1A and HNPP (hereditary neuropathy with pressure palsies) yielded negative results. CSF (cerebrospinal fluid) showed a protein level of 0.54 g/L, normal cell count and absent oligoclonal bands. MRI of the brain, cervical spine and both plexus brachialis showed normal findings.

The diagnosis of Lewis-Sumner syndrome (MADSAM) was made at the time (according to EFNS/PNS criteria 2010). The treatment started with methylprednisolone 4 g intravenously. Oral prednisone (1 mg/kg/day) continued for two months. No clinical improvement led us to start with immunoglobulin therapy (IVIG, 0.4 g/kg/day, five days every month). After the 3^rd^ cycle of IVIG, upper limb weakness transiently improved (right upper limb wrist extensors MRC 4, finger extensors MRC 3, left upper limb wrist extensors MRC 5 and finger extensors MRC 4). Despite the frequent and highly dosed IVIG therapy during the next two-year treatment period, weakness progression continued. Later, patient underwent three cycles of plasma exchange in period of six months (five exchanges in each cycle) without any clinical improvement. Finally, one dose of ocrelizumab (two 300 mg infusions) was administered off label as the last therapeutic attempt. The patient continued to progress despite ocrelizumab treatment (right wrist extensors MRC 1, finger extensors MRC 1, wrist and finger flexors MRC 2, intrinsic hand muscles MRC 2, left wrist extensors MRC 2, finger extensors MRC 1, wrist and finger flexors MRC 3, intrinsic hand muscles MRC 2). Moreover, weakness and muscle wasting spread to proximal upper limb muscles bilaterally (supinator, pronator, biceps, brachialis, deltoideus with MRC 4). Minimal weakness progression was also registered in the distal muscles of both lower limbs with (MRC 4). Therefore, in conclusion, intensive long-lasting immunomodulatory therapy was unsuccessful (Table 2).

**Table 2:** Clinical course and treatment of polyneuropathy in patient II/5

Time (age) course	Symptoms	INCAT [6] 0–5UL/ LL	iRODS [7] 0–48	MRC [8] 0–60UL/LL	Diagnosis/Therapy
5/2016 (56)	Isolated asymmetric distal weakness and paresthesias of right UL,Generalized hyporeflexia,Pes excavatus	1/0	46	28/30	Suspicion of CMT1A and HNPP(PMP22 negative)Vitamins,Rehabilitation recommended
11/2016 (56)	Progression of distal weakness of right UL,Generalized hyporeflexia,Pes excavatus	2/0	43	25/30	Suspicion of MADSAMIV methylprednisolone 4 g (no effect)
1/2017 (57)	Mild distal weakness and paresthesias of left UL,Moderate distal weakness of right UL,Areflexia,Pes excavatus	2/0	42	24/30	IVIG 2 g/kg(transitory improvement of muscle strength of UL after 3rd cycle of IVIG)Continuation of IVIG therapy 2 g/kg every 4 weeks for 6 months,Later 1 g/kg every 3 weeks
1/2019 (58)	Progression of distal muscle weakness and hypotrophy in small hand muscles and forearm muscles bilaterally	3/0	38	22/30	PE (3 cycles),IVIG 2 g/kg every 4 weeks
1/2020 (59)	Severe progression of distal weakness of both UL,Hypotrophy and weakness of forearm and proximal UL muscles,mild distal weakness of LL	4/1	35	20/28	Ocrelizumab

*HNPP* Hereditary neuropathy with tendency to pressure palsy, *MADSAM* Multifocal acquired demyelinating sensory-motor polyneuropathy, *UL* Upper limb, *LL* Lower limb, *PE* Plasma Exchange, *IVIG* Intravenous immunoglobulins, *INCAT* Inflammatory Neuropathy Cause and Treatment Disability Score, *IV* Intravenous, *iRODS* Inflammatory Rasch-built Overall Disability Scale, *MRC* Medical Research Council Sum Score

**Fig. 1:**
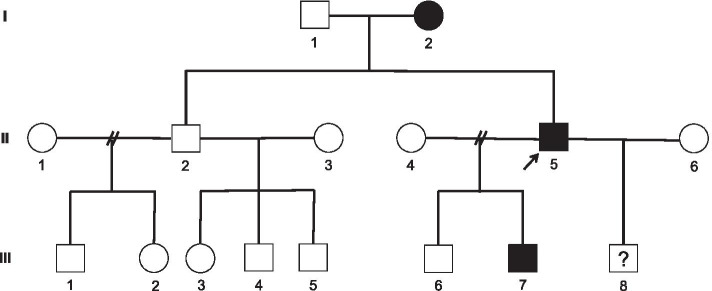
The three-generation pedigree of the family carrying a novel dominant variant c.348G > C of the *LITAF* gene (leading to amino acid substitution p.Trp116Cys). The proband (II.5) is marked with an arrow. All three affected individuals (I.2, II.5, and III.7) were heterozygotes carrying the recessive wt *LITAF* allele and the dominant mutated *LITAF* allele. The son (II.8, 2 years old) born in the second marriage of the proband has not yet been screened for the mutated *LITAF* allele (therefore labelled with a question mark). His probability of carrying the mutated allele c.348G > C was 50%

A survey of the patient´s family revealed signs of polyneuropathy in two other family members: the proband’s mother (79 years old, I.2) and his son (31 years old, III.7, Fig. 1). Both of them were asymptomatic (they did not complain about any motor or sensory disturbance in the upper or lower limbs). However, both of them had an evident bilateral pes cavus, generalized areflexia and decreased vibratory sense in the lower limbs. NCS revealed moderate primary demyelinating sensory-motor polyneuropathy of the upper limbs and severe demyelinating polyneuropathy of the lower limbs with partial conduction blocks and prolonged distal CMAP duration in some nerves (Table 3).

**Table 3:** Nerve conduction studies in son III/7 (31 years old)

Nerve	Nerve segment	11/2020 (dx)					11/2020 (sin)				
**MNCS**		Lat ms	Amp mV	MCV m/s	Dur ms	FW min lat ms	Lat ms	Amp mV	MCV m/s	Dur ms	FW min lat ms
**Median**											
Wrist	Wr-APB	5.7	11.0		8.8	NE	5.3	12.8	32.5	8.2	45.4
Elbow	El-Wr	13.0	7.7	31.6	10.2		12.8	9.1		10.4	
Axilla	Ax-El	19.2	6.1	45.5	11.3 12.1						
**Ulnar**											
Wrist	Wr-ADM	4.1	8.9	33.4	8.5	48.8	4.35	9.4	29.5	8.5	47.4
Elbow	BE-Wr	11.0	6.9	27.8	10.0		11.8	7.3	31.3	9.8	
Axilla	AE-BE	14.6	6.4	33.8	10.7		15.0	6.5		10.1	
	Ax-BE	21.4	5.1		11.8						
**Radial**											
Forearm	For-EIP	3.7	4.4	43.3	9.4		NA				
Elbow	CF-For	6.7	4.1	48.1	9.6						
Arm	Spir.g – CF	8.8	3.8	58.6	9.8						
		13.1	3.3		9.8						
**Peroneal**											
Ankle	An-EDB	10.7	0.03	22.9	6.9	NE	9.4	0.08	20.5	6.7	NE
Fibula	FH-An	25.1	0.21	23.3	7.2		25.5	0.18	22.7	6.9	
FP	FP-FH	29.2	0.16		7.3		29.9	0.14		7.2	
**Tibial**											
Ankle	AH-An	9.4	0.95	20.4	5.4	NE	6.2	1.8	21.0	7.7	NE
FP	An-FP	31.0	0.62		5.3		25.3	0.8		11.5	
**SNCS**		Lat ms	Amp uV	SCV m/s			Lat ms	Amp uV	SCV m/s		
**Median**	Wr-II	5.3	21.5	32.3			4.6	14.3	34.4		
**Ulnar**	Wr-V	4.4	14.3	32.1			4.5	18.7	33.3		
**Radial**	Wr-I	3.3	10.5	31.2			3.4	12	30.8		
**Sural**	Calf-LM	NE					NE				

Legend: *MNCS* Motor nerve conduction studies, *SNCS* Sensory nerve conduction studies, *Lat* Latency, *Amp* Amplitude, *Dur* Duration, *FW min* Minimal latency of F wave, *MCV* Motor conduction velocity, *SCV* Sensory conduction velocity, *AH* Abductor hallucis, *APB* Abductor pollicis brevis, *ADM* Abductor digiti minimi, *EDB* Extensor digitorum brevis, *EIP* Extensor indicis proprius, *Wr* Wrist, *AE* Above elbow, *BE* Below elbow, *For* Forearm, *Ax* Axilla, *CF* Cubital fossa, *Spir.g* Spiral groove, *An* Ankle, *FH* Fibular head, *FP* Fossa poplitea, *LM* Lateral malleolus, *NA* Not available, *NE* Not elicited, *ms* Millisecond, *uV* Microvolt, *m/s* Meter per second

**Fig. 2:**
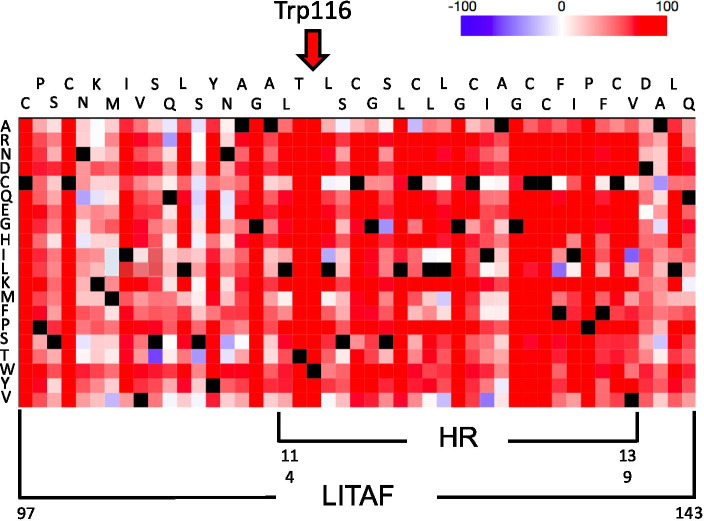
Amino acid substitutions shown independently for each amino acid position of the partial sequence of the LITAF protein (C97 – Q143) in the form of a heatmap generated by Predict Protein Open (Effect of Protein Mutations; https://predictprotein.org; Request ID: $1$pF7RZHuV$IeSzz1Fm8wUJ0HRi4TfLk). Red indicates a high score (score > 50, strong signal for effect), white indicates weak signals (-50 < score < 50), and blue indicates a low score (score < -50, strong signal neutral/no effect). Black marks the corresponding wt amino acid residues. The functional effects of the mutation are predicted with SNAP2, the trained classifier based on a neural network machine-learning device (https://predictprotein.org). The Trp residue present at position 116 of wt LITAF subjected to substitution with the Cys residue in the novel, mutated LITAF variant identified in our family is highlighted. The abbreviation HR stands for “hydrophobic region”

**Fig. 3:**
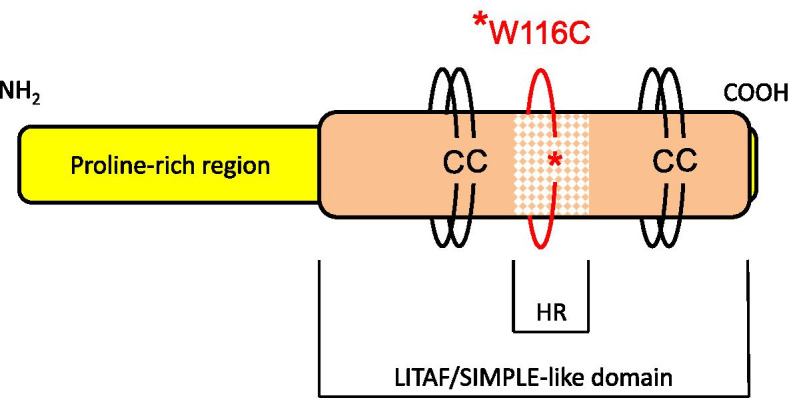
Graphic representation of LITAF protein organization. It is a 161-amino acid polypeptide with an MW of 17 kDa. The protein consists of a proline-rich region and a LITAF/SIMPLE-like domain. This domain responsible for the association of the protein with the membrane is divided into the left and right Cys-Cys (CC) arms, which are both separated by the 22 to 25 amino acid hydrophobic region (HR). In LITAF variant p.W116C, the Trp at position 116 has been substituted by Cys. The position of the substitution is on the very left end of the HR domain

The LITAF/SIMPLE (ENST00000339430) bidirectional sequencing of the proband’s sample (II.5, Fig. 1) showed a G > C transversion in exon 4 at position 348 (c.348G > C/p.Trp116Cys, Fig. 2 and Fig. 3) present in the heterozygous state. This novel, missense variant of LITAF had not been previously reported elsewhere and was scored as pathogenic by Predict Protein Open (Effect of Protein Mutations) software (Fig. 2) and other predictive software programs (GERP, MetaSVM, PolyPhen 2 and MutationTaster). Furthermore, the mutation was scored as 'pathogenic' by the ACMG (American College of Medical Genetics) criteria (PS1 + PM2 + PM5 + PP1 + PP2 + PP3 + PP4) [9]. No other plausible, causative candidate variants were found in the proband’s genetic material by WES (whole exome sequencing). WES results were verified by the amplicon deep sequencing (ADS) method. PCR amplicons (545 bp long) were generated using specific primers (forward: actggtgttccttccccttt, reverse: tcgtttgaacctgagcagtg). The amplicons were prepared for sequencing using a Nextera XT kit (Illumina) according to the manufacturer’s instructions. Libraries were paired-end (2 × 100 bp) sequenced on an Illumina HiSeq1500. The results were visualized on the Integrative Genomics Viewer and analysed for the presence of the particular variant in proband. The ADS verification resulted in obtaining coverage > 6000 × in all cases. The missense variant c.348G > C of LITAF was also identified in family members I.2 and III.7 which correlated with autosomal dominant pattern of inheritance (Fig. 1). Genetic analysis did not confirm the missense variant in healthy family members (father I.1, proband´s brother II.2, other son III.6).

## Discussion

CMT1C has been described as a symmetrical length-dependent mild form of slowly progressive sensory-motor demyelinating polyneuropathy, and lower limbs are affected more often. The clinical phenotype may differ between individuals even in one family with the same mutation in LITAF [4, 5]. The disease in our patient started as subacute asymmetric distal mild weakness and paraesthesia of the right upper limb. Clinical examination revealed pes excavatus, hyporeflexia and loss of vibration sense in the lower limbs. After a longer progression of weakness, the involvement became bilateral with a rapid deterioration of strength in both upper limbs and the development of proximal muscle weakness. Clinically, in addition to the stepwise or sudden onset of rapid progressive strength decline, the presence of pain and positive sensory symptoms as well as proximal weakness represent the “red flag” signs for an inflammatory component in CMT [10].

The uniformity of conduction slowing is a typical feature in most hereditary demyelinating neuropathies. However, CMT1C was previously described as a primary demyelinating polyneuropathy with conduction blocks (also patchy slowing and temporal dispersion) in some nerves [10, 11]. Such a finding may confuse differential diagnosis between hereditary and inflammatory neuropathy, as noted in our case. Increased distal CMAP duration, abnormal temporal dispersion and proximal motor conduction block are part of the electrodiagnostic criteria for CIDP (chronic inflammatory demyelinating polyneuropathy), but they were also reported in several inherited neuropathies [4, 11–14].

Occasionally, a patient with genetic neuropathy develops superimposed CIDP and improves with immune suppressants or modulatory medication [10, 15, 16]. When rapid deterioration is observed, even when an inflammatory component is questionable/unproven, the indication for immunotherapy appears to be justified [10]. In our case, we observed the patient’s poor response to long-lasting and intensive immunotherapy (steroids, IVIG, plasma exchange and ocrelizumab). A definite and undisputed therapeutic response is a major supportive criterion for a positive diagnosis of inflammatory neuropathy, e.g., CIDP [17]. In the case of treatment refractoriness, early consideration for tissue diagnosis and genetic testing should be realized [10]. The lack of benefit of immunotherapy together with the repeatedly negative results of auxiliary investigations (CSF, MRI and antibodies) and genetic testing (identification of the novel, pathogenic LITAF variant c.348G˃C/p. Trp116Cys (Fig. 3), which was also confirmed in two other family members), led us to the final diagnosis of hereditary CMT1C polyneuropathy with atypical clinical presentation.

There are several described cases of hereditary polyneuropathy, which were initially diagnosed as an inflammatory neuropathy, e.g., CIDP [13, 14, 18, 19]. The diagnostic discrimination between hereditary and inflammatory neuropathies is typically not an easy task. A subgroup of patients exhibit clinical features of both specific electrophysiological findings and variable responses to immunotherapy.

## Conclusions

The presentation of the CMT1C phenotype in our patient was unusual compared to patients with identical diagnoses in previous studies. This case substantiates the hypothesis that the phenotype related to CMT1C may have a wide spectrum of disease phenotypes and severities even in a single family comprising identical mutations. Therefore CMT1C should be added to the list of inherited polyneuropathies that need to be considered in suspected cases of inflammatory demyelinating neuropathy.

## Data Availability

All data generated or analysed during this study are included in this published article.

## Abbreviations

ACMG: American College of Medical Genetics
ADS: Amplicon deep sequencing
Anti-MAG antibodies: Antibodies against a myelin-associated glycoprotein
CIDP: Chronic inflammatory demyelinating polyneuropathy
CK: Creatine kinase
CMAPs: Compound muscle action potentials
CMT1A: Charcot-Marie-Tooth 1A
CMT1C: Charcot-Marie-Tooth 1C
CSF: Cerebrospinal fluid
HNPP: Hereditary neuropathy with tendency to pressure palsy
INCAT: Inflammatory Neuropathy Cause and Treatment Disability Score
iRODS: Inflammatory Rasch-built Overall Disability Scale,
IVIG: Intravenous immunoglobulins
LITAF: Lipopolysaccharide-induced tumour necrosis alpha factor
LL: Lower limb
MADSAM: Multifocal acquired demyelinating sensory and motor polyneuropathy
MRC: Medical Research Council
MRI: Magnetic resonance imaging
NCS: Nerve conduction studies
PE: Plasma exchange
SNAPs: Sensory nerve action potentials
UL: Upper limb
WES: Whole exome sequencing
